# Does acute-phase acupuncture at Hegu (LI04) promote facial nerve regeneration and upregulation of the PI3K/Akt/mTOR pathway? A hypothesis-generating study in a rabbit crush injury model

**DOI:** 10.3389/fneur.2026.1805719

**Published:** 2026-04-22

**Authors:** Jin Song, Jianyun Zhang, Xinsheng He, Zhichun Zhu, Bingyan Liu, Yang Liu, Chen Liang, Binbin Jiang, Kesheng Zhang

**Affiliations:** Rehabilitation and Treatment Center, Hunan Rehabilitation Hospital, Changsha, China

**Keywords:** acupuncture, facial nerve injury, Hegu (LI04), intervention timing, nerve regeneration, PI3K/Akt/mTOR pathway

## Abstract

This study used the rabbit facial nerve crush injury model to investigate the time-dependent effects of acupuncture at Hegu (LI04) on facial nerve regeneration and regulatory role of the phosphatidylinositol 3-kinase (PI3K)/protein kinase B (Akt)/mammalian target of rapamycin (mTOR) pathway. Thirty-two New Zealand rabbits with facial nerve crush injury were randomly assigned to four groups (*n* = 8 each): sham-operated, model, acute-phase acupuncture (treatment initiated immediately after modeling, daily for 10 days), and chronic-phase acupuncture (treatment initiated 7 days after modeling, daily for 10 days). Simone’s 10-point scale was used for behavioral assessment on day 1 and day 17 post-modeling. Hematoxylin & Eosin staining was used for histomorphology analysis. PI3K, Akt, and mTOR protein and mRNA expression levels were assessed by immunohistochemistry and RT-qPCR, respectively. Both acute- and chronic-phase acupuncture interventions significantly improved the facial nerve function scores, reduced myelin sheath edema, and upregulated the expression levels of PI3K, Akt, and mTOR compared to the model group. Compared to the chronic-phase intervention, the acute-phase intervention yielded superior functional recovery, more regular nerve fiber arrangement, and stronger upregulation of PI3K, Akt, and mTOR proteins and mRNAs. In this experimental model, acupuncture at Hegu (LI04) was associated with improved repair of facial nerve crush injury, with acute-phase intervention showing greater efficacy than chronic-phase intervention. This time-dependent effect was accompanied by upregulation of the PI3K/Akt/mTOR signaling pathway, suggesting a potential association. However, given the substantial methodological limitations—including the absence of sham controls, lack of electrophysiological validation, and the partial nature of the injury model—these findings provide preliminary experimental evidence that may help inform the design of future clinical studies, rather than serving as confirmatory evidence.

## 1 Introduction

Peripheral facial paralysis is a common neurological disorder that is primarily manifested as facial nerve injury. It is the sixth most prevalent neurological disease in China, affecting approximately 3 million individuals annually (1). Peripheral facial paralysis, particularly idiopathic facial palsy (Bell’s palsy), has a well-documented natural history with approximately 70–85% of patients experiencing spontaneous recovery within months (2, 3). Systemic corticosteroid therapy remains the only widely accepted evidence-based treatment in the acute phase and significantly improves recovery outcomes when administered early (4, 5). Current clinical guidelines, including those from the American Academy of Neurology (4) and the Japan Society of Facial Nerve Research (5), consistently recommend early corticosteroid therapy as the standard of care for acute Bell’s palsy.

Despite this, some patients seek complementary therapies such as acupuncture, which is commonly used in several Asian countries (6, 7). However, clinical evidence regarding the efficacy and optimal timing of acupuncture remains limited and conflicting: a recent umbrella review concluded that high-quality evidence supporting physical therapies, including acupuncture, for Bell’s palsy is insufficient (3). While some studies suggest that early acupuncture intervention may shorten recovery time (8, 9), others identify acupuncture as a negative prognostic factor (10). This discrepancy highlights the need for mechanistic investigations to explore the time-dependent effects of acupuncture and its potential biological pathways, rather than assuming clinical efficacy.

The phosphatidylinositol 3-kinase (PI3K)/protein kinase B (Akt)/mammalian target of rapamycin (mTOR) signaling pathway is a critical regulator of neuronal survival, axonal growth, and myelin repair, and its direct activation is sufficient to promote facial nerve regeneration (11). Acupuncture facilitates nerve repair by modulating pro-regenerative pathways such as PI3K/Akt/mTOR (12). Previous studies suggest that acupuncture exerts neuroprotective effects by modulating the PI3K/Akt/mTOR pathway (13, 14), but these studies did not focus on Hegu (LI04) or systematically compare the effects of acupuncture in acute phase versus the chronic phase. Specifically, previous studies have several limitations: (1) most focused on local acupoints without assessing core pro-regenerative pathways (8, 9); (2) studies that did examine the PI3K/Akt/mTOR pathway did not specifically target Hegu (LI04) (13, 14); and (3) no study has systematically compared acute-phase versus chronic-phase acupuncture at Hegu (LI04) in a standardized facial nerve crush injury model while exploring the underlying molecular mechanisms.

Therefore, in this study, we used the well-established rabbit facial nerve crush injury model to systematically compare the therapeutic efficacy of acute-phase versus chronic-phase manual acupuncture at Hegu (LI04)—a classic distant acupoint with proven clinical efficacy for head and facial disorders (15). We also investigated its association with modulation of the PI3K/Akt/mTOR pathway, a critical regulator in facial nerve regeneration (11). Our aim was to generate hypothesis-generating evidence regarding the time-dependent effects of Hegu (LI04) acupuncture, which may inform the design of future clinical studies investigating optimal intervention timing.

## 2 Materials and methods

### 2.1 Experimental animals and ethics

Thirty-two healthy male New Zealand White rabbits (6–8 months old, weighing 2.50 ± 0.25 kg) were purchased from the Laboratory Animal Center of Hunan University of Traditional Chinese Medicine. Animals were housed individually in standard rabbit cages (60 cm × 50 cm × 40 cm) under controlled conditions: temperature 22 ± 2 °C, humidity 50–60%, 12-h light/dark cycle (lights on at 7:00 a.m.). Food and water were available ad libitum. This animal study was approved by the Animal Ethics Committee of Hunan University of Traditional Chinese Medicine (Approval No. HNUCMH21-2407-06). All procedures were conducted under anesthesia in accordance with relevant international guidelines and the 3Rs principles, and efforts were made to minimize animal suffering (Animal license: SYXK(Xiang) 2024-0014).

### 2.2 Animal randomization and grouping

The rabbits were randomly assigned to one of the following four groups (*n* = 8 each) using a computer-generated random number table: sham-operated, model, acute-phase acupuncture, and chronic-phase acupuncture.

### 2.3 Facial nerve crush injury model

The facial nerve crush injury model was established as previously described (16), with slight modifications. Under general anesthesia induced by intravenous pentobarbital sodium (30 mg/kg) administered via the marginal ear vein, the right buccal branch of the facial nerve was exposed through a 2-cm vertical incision posterior to the mandible. The buccal branch was identified by its anatomical course superficial to the masseter muscle and carefully dissected from surrounding connective tissue. The buccal branch was selected because it is easily accessible and commonly used in studies investigating localized nerve repair mechanisms; however, it produces a partial, branch-specific injury that may not recapitulate the global facial paralysis seen in clinical practice. A standardized crush injury was created by clamping the nerve 5 mm distal to the parotid gland with a mosquito forceps (Chinese medical device registration number: Su Huai Xie Bei 20220032, length 140 mm) closed to the first ratchet for exactly 5 min, producing a 1.5 mm lesion. The forceps had a smooth jaw surface to avoid nerve transection. The incision was closed in layers with 5–0 silk sutures. All surgeries were performed by the same experienced operator (C. L.), an otorhinolaryngologist with 10 years of surgical experience, to ensure consistency. Anesthesia depth was monitored by pedal reflex and respiratory rate. Postoperative analgesia was provided by subcutaneous buprenorphine (0.05 mg/kg) every 12 h for 48 h. Successful modeling was confirmed by a facial nerve motor function score of <4 on the Simone 10-point scale (17). The sham-operated group underwent identical surgical exposure without nerve clamping. Postoperative care included daily monitoring of wound healing, weight, and general behavior.

### 2.4 Acupuncture intervention

Bilateral Hegu (LI04) was selected based on the Traditional Chinese Medicine meridian theory. Manual acupuncture was chosen over electroacupuncture because it is more direct and the stimulation intensity can be controlled (18). All acupuncture procedures were performed by a licensed acupuncturist (Z. Z.) with 20 years of clinical experience and 3 years of experience in animal acupuncture research, ensuring standardized and reproducible needle manipulation. Disposable stainless-steel needles (0.25 mm × 25 mm) were inserted perpendicularly to a depth of 8 mm at LI04. The even reinforcing-reducing technique was applied. The needles were retained for 20 min. The achievement of Deqi was operationally defined by rhythmic local muscle twitching in the ipsilateral paw upon needle manipulation, a commonly used behavioral indicator in rabbit acupuncture models (18). The acute-phase acupuncture group received daily treatment immediately after successful modeling for 10 consecutive days. The chronic-phase acupuncture group started treatment on day 7 post-modeling, with the same needling protocol, for 10 consecutive days. The sham-operated and model groups did not receive any acupuncture intervention. Final behavioral assessment was performed on day 17 post-modeling for all groups.

### 2.5 Sample collection

After the final behavioral assessment, all rabbits were euthanized under deep anesthesia by intracardiac injection of pentobarbital sodium (100 mg/kg). The injured facial nerve segment was carefully dissected and harvested. Samples were either fixed in 4% paraformaldehyde for histology or snap-frozen in liquid nitrogen for molecular analysis.

### 2.6 Outcome assessments

#### 2.6.1 Facial nerve function evaluation

Facial nerve function was evaluated using the Simone 10-point scale on day 1 (post-modeling) and day 17 (post-modeling) by two independent investigators (B. L. and Y. L.) who were blinded to group allocation. The scores from the two investigators were averaged for analysis.

#### 2.6.2 Histomorphological observation

The injured facial nerve segments were fixed, embedded in paraffin, sectioned, and stained with hematoxylin and eosin (H&E). The arrangement of nerve fibers, axonal integrity, and myelin sheath status were observed under a light microscope. For histomorphological analysis, three rabbits per group were randomly selected using a random number table. Three sections per animal were examined, and the most representative images were chosen for presentation.

#### 2.6.3 Immunohistochemistry

IHC was used to detect the protein expression levels of PI3K, Akt, and mTOR. The average optical density (OD) of positive staining was quantified using the Image-Pro Plus 6.0 software.

#### 2.6.4 RT-qPCR analysis

Total RNA was extracted from the harvested facial nerve tissue segment (the crushed region and adjacent area). cDNA was synthesized from 1 μg of total RNA using the ExonScript RT SuperMix with dsDNase kit (Exongen, catalog no. A502). The 20 μL reaction, containing 4 μL of 5 × Reaction Buffer and 3 μL of Supreme Enzyme Mix, was incubated at 25 °C for 10 min (gDNA removal), 55 °C for 15 min (reverse transcription), and 85 °C for 5 min (enzyme inactivation). The cDNA was diluted and stored at −20 °C. Quantitative PCR was performed using SYBR Green PCR Master Mix (Applied Biosystems, catalog no. 4472920) on a Longgene Q2000B Real-Time PCR System. Each 20 μL reaction contained 10 μL of Master Mix, 0.4 μL each of forward and reverse primer (10 μM, sequences in Table 1), and 2 μL of cDNA template. The thermal profile was: 95 °C for 5 min; 40 cycles of 95 °C for 10 s, 58 °C for 20 s, 72 °C for 20 s; followed by a melt-curve analysis. Gene expression was calculated using the 2^−ΔΔCt^ method with β-actin as the reference. Primer sequences are listed in Table 1.

**Table 1:** Primer sequences.

Target gene	Primer sequence	Product length (bp)
Actin	F: ACGACATGGAGAAGATCTGGCAC	142
	R: AACGTCTCGAACATGATCTGGGT	
PI3K	F: TGATGCAGCCATTGACCTGT	194
	R: GTGCCGATCTCCAATTCCCA	
Akt	F: AACCAAGCCCACATCTCCTG	148
	R: AGACGGCTGCACATAGACACAC	
mTOR	F: CCCGCCATTTTGGAGTCTCT	110
	R: TGGATCTCCAGCTCTCCGAA	

### 2.7 Statistical analysis

Statistical analysis was performed using the IBM SPSS Statistics 29.0 software. All measurement data were presented as mean ± standard deviation (*x̄* ± *s*). One-way analysis of variance (one-way ANOVA) was used for inter-group comparisons when the data met assumptions of normality and homogeneity of variances, followed by the least significant difference (LSD) test for pairwise comparisons. If the variance was heterogeneous, Tamhane’s T2 test was applied. A two-tailed *p*-value < 0.05 was considered statistically significant. Sample size was determined based on previous studies using similar animal models. Fei et al. (14) used 6–8 rabbits per group at each time point in a facial nerve crush injury model and reported significant differences between treatment groups. However, no formal *a priori* power analysis was performed to calculate the required sample size. This is a limitation, as it may affect the statistical robustness of the findings and increases the risk of type II errors.

## 3 Results

### 3.1 Comparison of behavioral scores

**Figure 1:**
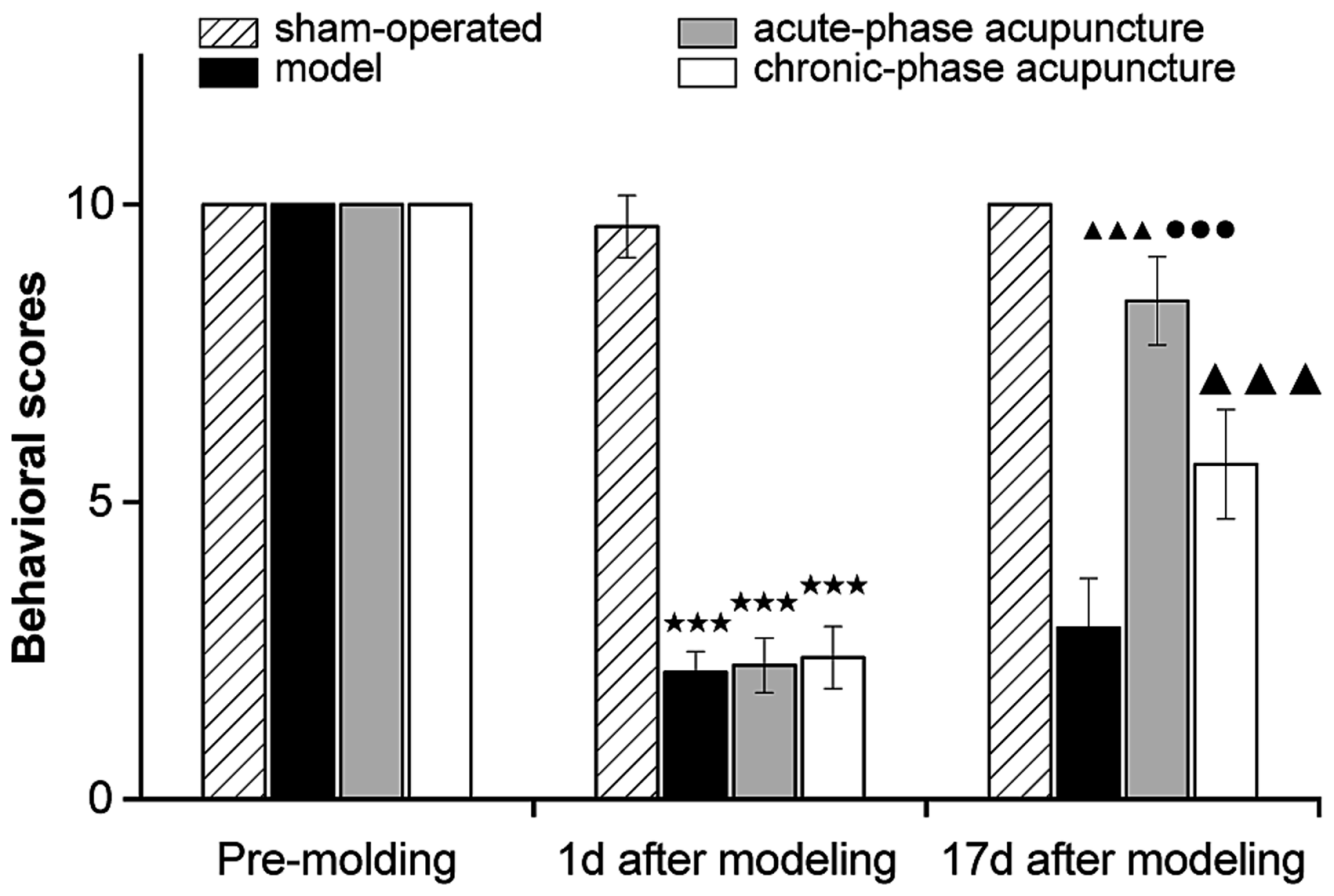
Facial nerve function scores of rabbits in each group (*n* = 8). Data are expressed as mean ± SD. ^★^*p* < 0.05, ^★★^*p* < 0.01, ^★★★^*p* < 0.001 vs. Sham; ^▲^*p* < 0.05, ^▲▲^*p* < 0.01, ^▲▲▲^*p* < 0.001 vs. Model; ^●^*p* < 0.05, ^●●^*p* < 0.01, ^●●●^*p* < 0.001 vs. Chronic-phase group.

Before modeling, there were no significant differences in behavioral scores between the groups (*p* > 0.05; Figure 1). On day 1 post-modeling, behavioral scores for the model, acute-phase, and chronic-phase groups were significantly lower than the sham group (*p* < 0.001), thereby indicating that injury severity was comparable initially. After treatment (day 17), both acupuncture groups showed significantly higher facial nerve function scores compared to the model group (*p* < 0.001). Furthermore, the acute-phase acupuncture group demonstrated significantly higher functional recovery than the chronic-phase acupuncture group (*p* < 0.001).

### 3.2 Histomorphology analysis of facial nerve by H&E staining

**Figure 2:**
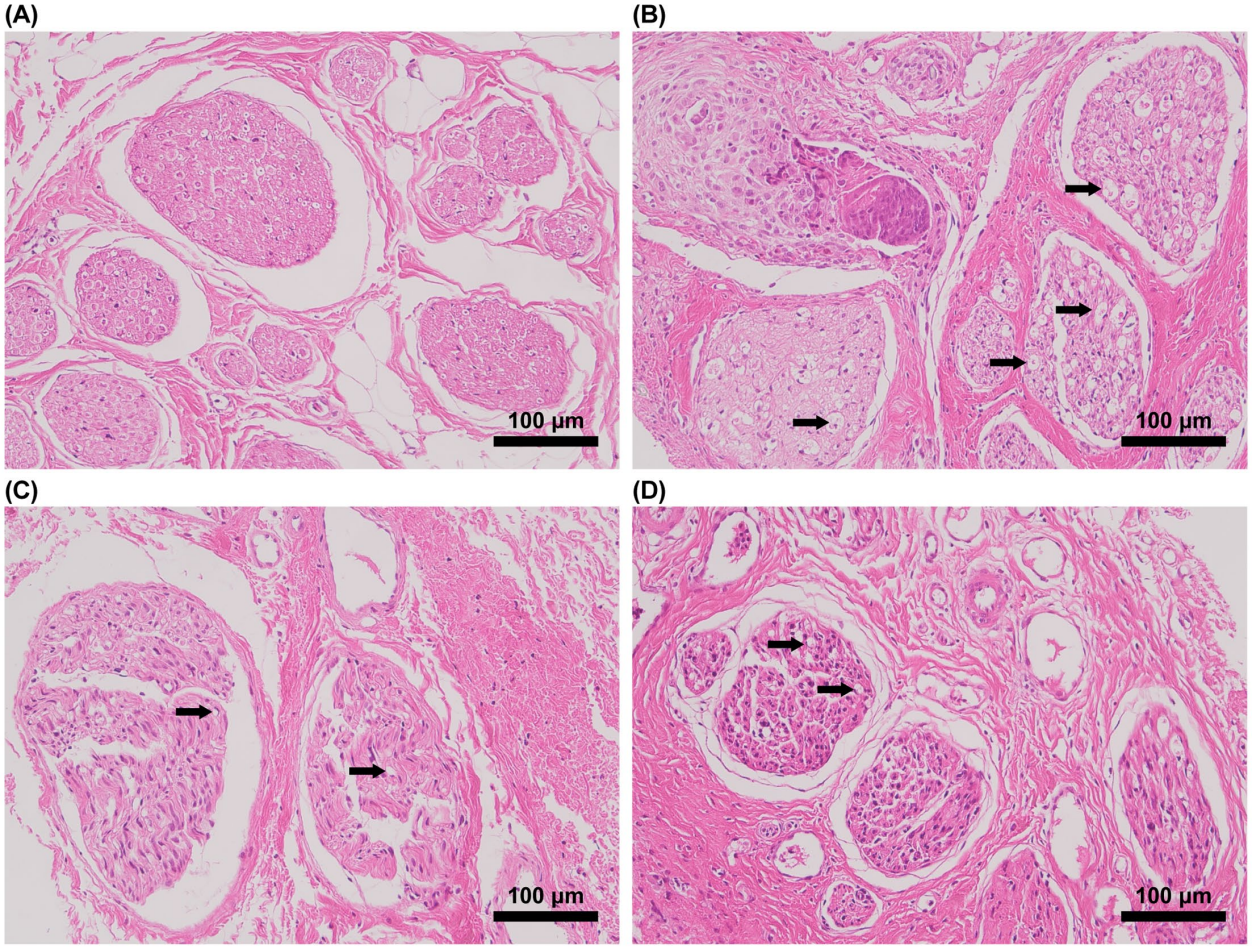
Morphological results of H&E staining of rabbit facial nerves (*n* = 3, scale bar = 100 μm). **(A)** Sham-operated group, **(B)** Model group, **(C)** Acute-phase acupuncture group, and **(D)** Chronic-phase acupuncture group. Arrows indicate areas of edema **(B,D)** and heterochromatic bodies **(B)**.

H&E staining results demonstrated neatly arranged nerve fibers, intact axons, and normal myelin sheaths in the sham group (Figure 2). The model group exhibited disorganized nerve fibers, significant myelin sheath edema, and occasional infiltration of inflammatory cells. The acute-phase acupuncture group showed more regular nerve fiber arrangement and reduced edema compared to both the model and chronic-phase groups. The chronic-phase group demonstrated considerable structural disorganization and edema.

### 3.3 Immunohistochemistry analysis of PI3K, Akt, and mTOR protein levels

**Figure 3:**
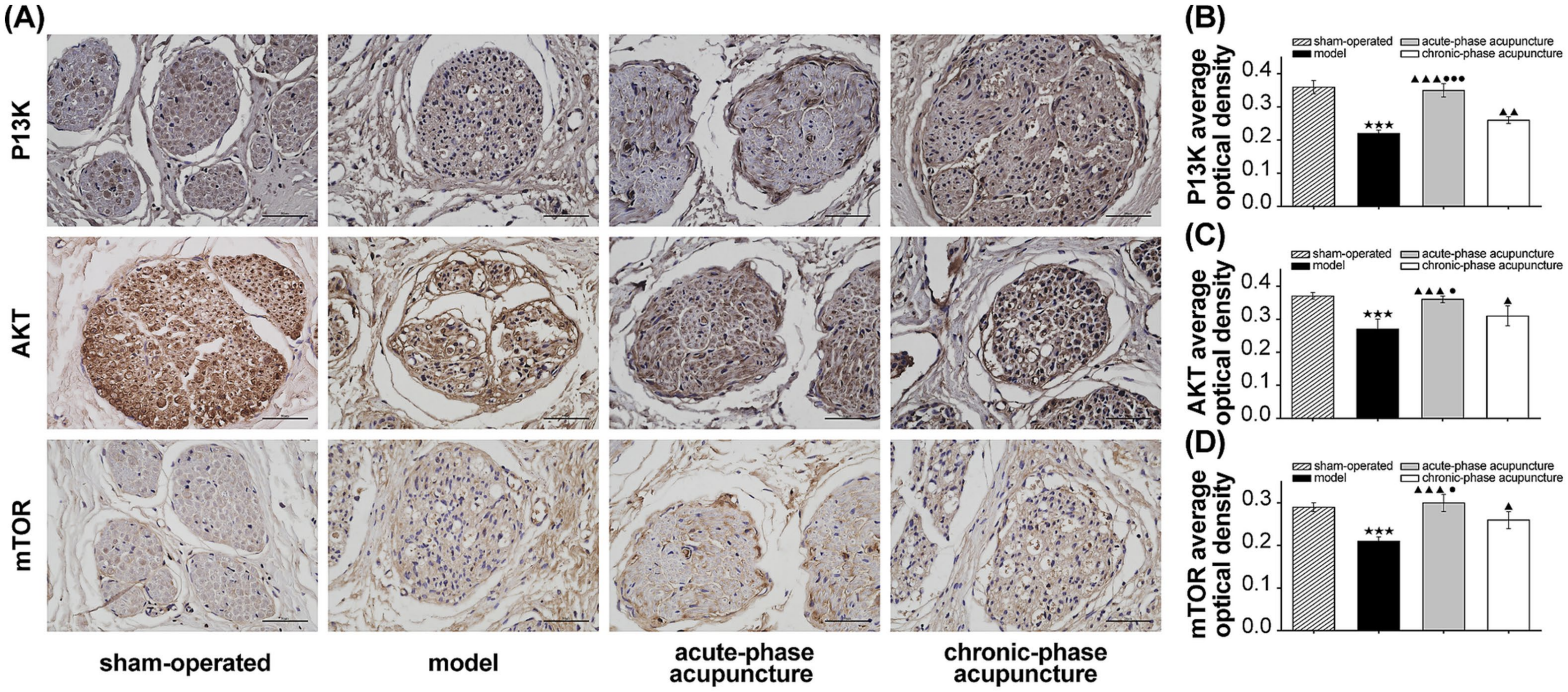
**(A)** Representative immunohistochemical staining of PI3K, Akt, and mTOR in the facial nerve tissues from different groups (*n* = 3, scale bar = 50 μm). **(B–D)** Quantitative analysis of the average optical density (OD) for PI3K, Akt, and mTOR protein expression. Data are expressed as mean ± SD. ^★^*p* < 0.05, ^★★^*p* < 0.01, ^★★★^*p* < 0.001 vs. Sham; ^▲^*p* < 0.05, ^▲▲^*p* < 0.01, ^▲▲▲^*p* < 0.001 vs. Model; ^●^*p* < 0.05, ^●●^*p* < 0.01, ^●●●^*p* < 0.001 vs. Chronic-phase group.

The expression levels of PI3K, Akt, and mTOR proteins were significantly lower in the model group compared to the sham group (*p* < 0.001; Figure 3). Both acupuncture groups showed significantly higher expression levels of these proteins compared to the model group. Furthermore, the acute-phase acupuncture group exhibited significantly higher expression levels of PI3K, Akt, and mTOR proteins than the chronic-phase group (*p* < 0.001, *p* < 0.05).

### 3.4 RT-qPCR analysis of PI3K, Akt, and mTOR mRNA levels

**Figure 4:**
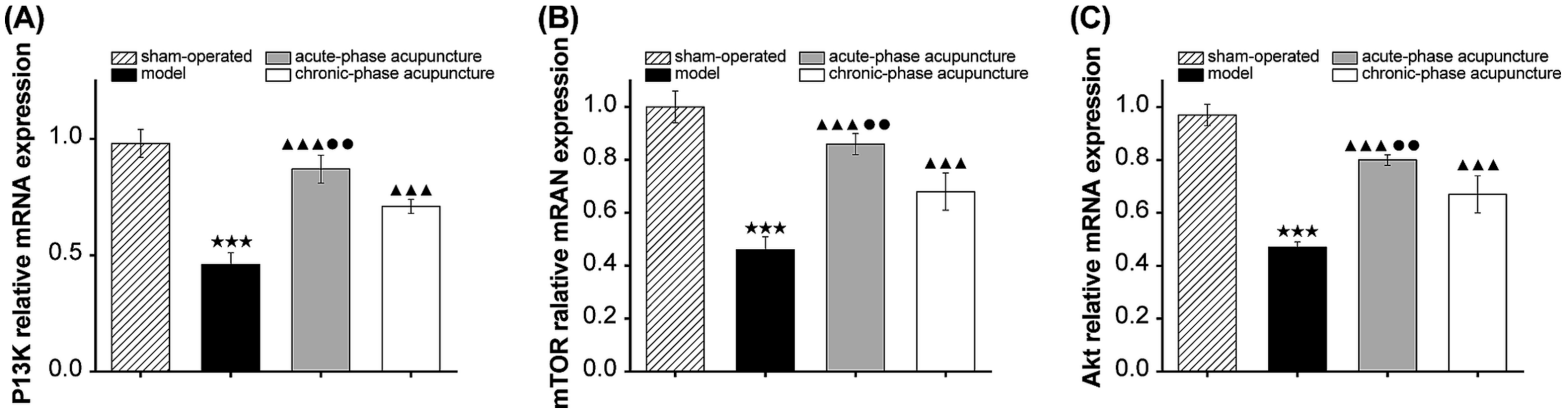
Relative mRNA expression levels of **(A)** PI3K**, (B)** Akt, and **(C)** mTOR in the facial nerve tissues from different groups (*n* = 3). Data are expressed as mean ± SD. ^★^*p* < 0.05, ^★★^*p* < 0.01, ^★★★^*p* < 0.001 vs. Sham; ^▲^*p* < 0.05, ^▲▲^*p* < 0.01, ^▲▲▲^*p* < 0.001 vs. Model; ^●^*p* < 0.05, ^●●^*p* < 0.01, ^●●●^*p* < 0.001 vs. Chronic-phase group.

The relative expression levels of PI3K, Akt, and mTOR transcripts were significantly lower in the model group than in the sham group (*p* < 0.001; Figure 4). Acupuncture intervention significantly upregulated the mRNA expression levels of PI3K, Akt, and mTOR in both treatment groups compared to the model group (*p* < 0.001). The acute-phase acupuncture group demonstrated significantly higher mRNA levels of PI3K, Akt, and mTOR compared to the chronic-phase group (*p* < 0.01).

## 4 Discussion

The present study investigated the time-dependent effects of acupuncture at Hegu (LI04) on facial nerve regeneration and the involvement of the phosphatidylinositol 3-kinase (PI3K)/protein kinase B (Akt)/mammalian target of rapamycin (mTOR) signaling pathway in a rabbit model of facial nerve crush injury. The results demonstrated that acupuncture at Hegu (LI04) was associated with significantly improved functional recovery and morphological repair of the facial nerve, with superior efficacy in the acute phase (immediately post-modeling) compared to the chronic phase (7 days post-modeling). This therapeutic effect was associated with the upregulation of PI3K, Akt, and mTOR protein and mRNA levels, suggesting potential involvement of the PI3K/Akt/mTOR pathway in Hegu (LI04)-mediated facial nerve repair.

Our study uniquely combines Hegu (LI04) acupuncture with the PI3K/Akt/mTOR pathway to address a research gap, building on the results of previous studies while overcoming their limitations. Previous studies investigated the efficacy of local acupoints without assessing the effects on the core pro-regenerative pathway (8, 9), or investigated the effects of non-specific acupuncture interventions on the PI3K/Akt/mTOR pathway (13, 14). Furthermore, previous research studies did not systematically compare the acute versus chronic phase effects of Hegu (LI04) acupuncture—an acupoint with unique meridional connections to head and facial regions—on facial nerve regeneration via the PI3K/Akt/mTOR pathway in a standardized crush injury model. The integration of acupoint selection (Hegu, LI04), pathway analysis (PI3K/Akt/mTOR), and timing comparison (acute vs. chronic) represents the core innovation of this study, distinguishing it from existing literature.

The PI3K/Akt/mTOR pathway is a central regulator of neuronal survival, axonal growth, and myelin repair (19). Direct activation of the PI3K/Akt/mTOR pathway is sufficient to promote facial nerve regeneration (11). Acupuncture exerts neuroprotective effects by modulating this pathway and attenuating neuroinflammation (12). We acknowledge that facial nerve regeneration involves other mechanisms such as inflammatory modulation and Schwann cell activation (16), and acupuncture induces multi-dimensional biological effects on peripheral facial paralysis (7). Recent real-world evidence further supports the clinical benefit of acute-phase acupuncture-moxibustion intervention for idiopathic facial palsy (20), aligning with our experimental findings on the superiority of early treatment. The overall clinical efficacy and safety of acupuncture for this condition has been demonstrated in a recent overview of systematic reviews and meta-analyses (21). This robust clinical evidence provides a crucial context for interpreting our mechanistic findings regarding the PI3K/Akt/mTOR pathway and timing of the intervention. The present study focused on the time-sensitive component by investigating the PI3K/Akt/mTOR pathway—one of the most well-characterized core pro-regenerative pathways for facial nerve repair—using Hegu (LI04) acupuncture in a standardized model. Furthermore, emerging neuroimaging research suggests that acupuncture for acute-stage Bell’s palsy may also involve central regulatory mechanisms, as evidenced by fMRI studies (22), indicating a complex multi-level mode of action that warrants further integration with peripheral pathway analyses.

While the precise neuroanatomical mechanism linking LI04 stimulation to facial nerve regeneration remains speculative and is not directly supported by experimental evidence from this study, the selection of Hegu (LI04) in this study was based on both traditional meridian theory and emerging neurophysiological evidence (15). From a modern neuroanatomical perspective, Hegu is located in the first dorsal interosseous muscle and is innervated by the superficial branch of the radial nerve (C5–T1). Stimulation at this site may trigger somatosensory afferent input that projects to the spinal cord, brainstem, and supraspinal centers, potentially modulating autonomic and neuroendocrine responses (23). Recent neuroanatomical studies have demonstrated that acupuncture at distal limb points can activate specific autonomic pathways—such as the vagal-adrenal axis—through somatotopically organized neural circuits, thereby influencing systemic physiological functions (23). This framework is presented as a hypothesis-generating conceptual model to help interpret the systemic effects of distal acupoint stimulation, rather than as a demonstrated mechanism.

The consistency between total protein upregulation (IHC) and mRNA overexpression (RT-PCR) in our study aligns with the research paradigm of previous acupuncture-related neuroprotection studies (13, 14). For example, Chen (13) demonstrated that tube moxibustion regulated the PI3K/Akt pathway in rabbits with facial nerve injury by assessing total protein levels, and their findings were validated by functional and morphological improvements—similar to our findings. Fei et al. (14) also reported that electroacupuncture promotes facial nerve regeneration via the GDNF/PI3K/Akt pathway but the study did not evaluate phosphorylated protein levels. These studies collectively indicate that consistent upregulation of total protein and mRNA, combined with therapeutic outcomes, provides indirect evidence that suggests the involvement of the PI3K/Akt/mTOR pathway in our study. However, these findings do not demonstrate pathway activation, as phosphorylated forms of these proteins were not assessed. Given the absence of phosphorylated protein data, the mechanistic interpretation presented here should be considered exploratory and hypothesis-generating, rather than confirmatory.

While our study focused on the PI3K/Akt/mTOR pathway, we recognize that facial nerve regeneration is a complex process involving multiple interacting mechanisms. Acupuncture may also exert neuroprotective effects through anti-inflammatory pathways, such as inhibiting NF-κB and reducing pro-inflammatory cytokines (12). Additionally, acupuncture has been shown to promote Schwann cell proliferation and migration, which are critical for Wallerian degeneration and subsequent axonal regeneration (24). Emerging evidence also suggests that autophagy, a cellular degradation process, plays a role in nerve repair and may be modulated by acupuncture (25). Future studies should investigate whether Hegu (LI04) acupuncture regulates these processes and explore potential crosstalk between the PI3K/Akt/mTOR pathway and other signaling networks. Multi-omics approaches (e.g., proteomics, transcriptomics) could provide a more comprehensive understanding of the mechanisms underlying acupuncture-induced nerve regeneration.

Our findings suggest a possible molecular basis underlying the clinical effects of Hegu (LI04) acupuncture, thereby addressing a key research hotspot in this area of research (26). Furthermore, our results provide high-level evidence for the mechanisms underlying the effects of acupuncture (27). The current clinical guidelines reflect an evidence gap, leading to sub-optimal recommendations for acupuncture (5). Independent methodological reviews have highlighted limitations in the rigor of some acupuncture trials (28). Therefore, our findings provide experimental support for the molecular basis of the effects of Hegu (LI04) acupuncture. Hegu (LI04) is a classic acupoint for head and facial disorders, with documented effects for improving local microcirculation and neural excitability (15). This study suggests that acupuncture at Hegu (LI04) may be associated with inhibition of neuronal apoptosis, reduction of myelin sheath edema, and promotion of axonal regeneration in association with upregulation of the PI3K/Akt/mTOR pathway.

The superior efficacy of acute-phase intervention can be attributed to timely modulation of the post-injury microenvironment. After crush injury, the facial nerve undergoes acute degeneration within the first few days (16). Early Hegu (LI04) acupuncture may confer a dual advantage of inhibiting secondary degeneration and promoting repair by rapidly activating the PI3K/Akt/mTOR pathway and mitigating neuroinflammation (12). This coordinated action generates a favorable microenvironment for nerve regeneration and prevents irreversible tissue damage. In contrast, chronic-phase intervention begins after establishment of secondary degeneration and inflammation, thereby resulting in weaker pathway regulation and suboptimal recovery. This experimental advantage is corroborated by findings from clinical meta-analyses focusing on timing, which reported that acute-phase treatment shortens recovery time and reduces sequelae (28). Furthermore, real-world data analysis demonstrates that early intervention significantly lowers recurrence risk, thereby highlighting its long-term clinical value (29). These clinical observations align with a recent systematic review and meta-analysis, which offers high-level evidence for the superior efficacy of acute-phase acupuncture intervention (30).

This study has several limitations that should be considered. First, the sample size of eight rabbits per group is relatively small, and no formal power analysis was conducted prior to the experiment. Although this scale is consistent with that commonly used in exploratory mechanistic animal studies, and the reliability of the findings was ensured through strict randomization, blinded assessment, and appropriate statistical tests, a larger sample size would further strengthen the statistical power and generalizability of the results. The lack of power analysis means that we cannot rule out the possibility of type II errors (i.e., failing to detect a true effect). The consistent outcomes across behavioral, histomorphological, and molecular assessments reinforce the robustness of the conclusions; however, future studies would benefit from an increased sample size and should include formal power analysis to determine optimal sample size.

Second, due to time and resource constraints, we did not perform Western blotting analysis of phosphorylated proteins (p-PI3K, p-Akt, and p-mTOR). Nevertheless, consistent upregulation of total protein and mRNA levels, combined with functional and morphological improvements, provides indirect evidence that suggests the involvement of the PI3K/Akt/mTOR pathway (13, 14). However, these findings do not demonstrate pathway activation. Future studies will prioritize phosphorylated protein detection to validate pathway involvement.

Third, we did not perform electrophysiological assessments such as nerve conduction studies or electromyography, which are considered essential for objectively confirming nerve injury and monitoring functional recovery, particularly in partial injury models where behavioral scoring may be unreliable. Although behavioral scoring and histomorphology are widely accepted in exploratory animal studies, the absence of electrophysiological data critically limits the objective validation of our model and the functional significance of the observed improvements. In this study, we cannot rule out the possibility that compensatory mechanisms from adjacent nerve branches contributed to the observed behavioral recovery. Therefore, our findings should be considered preliminary and hypothesis-generating, and future studies must incorporate electrophysiological measures to confirm these observations.

Fourth, our experimental design did not include sham acupuncture, non-acupoint stimulation, or a corticosteroid treatment group. This is a critical methodological limitation, as it fundamentally prevents us from determining whether the observed effects are specific to the LI04 acupoint, reflect non-specific needle stimulation, or result from general physiological responses to tissue manipulation. While our primary aim was to compare the timing of acupuncture intervention, the absence of these controls means that this study cannot establish acupoint-specific efficacy. Therefore, our findings should be interpreted as demonstrating an association between needle stimulation at LI04 and the measured outcomes, without claiming acupoint specificity. Future studies incorporating these control groups are essential to validate the specificity of Hegu (LI04) acupuncture.

Fifth, the histological analysis was performed on a subset of animals (*n* = 3 per group). Although this is common practice in exploratory studies and the findings were consistent with functional and molecular results, increasing the sample size for histology in future confirmatory studies would enhance statistical power.

Sixth, the present study only included acute and chronic phases. The absence of a subacute phase (3 days post-modeling) limits precise definition of the “optimal intervention window,” but this aspect will be addressed in subsequent studies. Moreover, downstream targets of the PI3K/Akt/mTOR pathway (e.g., autophagy, apoptosis, inflammation) were not evaluated in this study. Therefore, future research should investigate whether Hegu (LI04) acupuncture regulates these biological processes to construct a more comprehensive mechanistic network.

We also acknowledge that using the buccal branch rather than the main trunk of the facial nerve substantially limits the generalizability of our findings to complete facial paralysis. The buccal branch was selected because it is easily accessible and commonly used in studies investigating localized nerve repair mechanisms; however, it produces a partial, branch-specific injury that may not recapitulate the global facial paralysis seen in clinical practice. The buccal branch primarily innervates the perioral muscles, and functional recovery was assessed using Simone’s 10-point scale, which includes multiple facial regions (eyelid closure, nose twitching, whisker movement, and lip position). In a partial injury model, behavioral scoring may not reliably reflect true functional recovery, as compensatory mechanisms from adjacent branches could influence the observed outcomes. Although we observed consistent deficits across multiple facial regions in our model, possibly due to the anatomical interconnection of facial nerve branches in rabbits (31), these findings should be interpreted as preliminary observations in a branch-specific injury model rather than as evidence for functional recovery in complete facial paralysis. Nonetheless, future studies using main trunk injury models and more refined functional assessments (e.g., video-based motion analysis, electrophysiology) would be valuable.

## 5 Conclusion

The present study provides preliminary experimental evidence suggesting that acupuncture at Hegu (LI04) may be associated with enhanced repair of facial nerve crush injury in rabbits, with acute-phase intervention showing potentially greater functional and morphological recovery compared to chronic-phase intervention. This time-dependent effect was accompanied by upregulation of PI3K, Akt, and mTOR proteins and transcripts, which may suggest involvement of the PI3K/Akt/mTOR signaling pathway. However, given the substantial methodological limitations discussed above—including the absence of sham controls, lack of electrophysiological validation, and the partial nature of the injury model—these findings should be considered strictly hypothesis-generating and exploratory. They do not establish a causal relationship between LI04 acupuncture and nerve regeneration, nor do they demonstrate pathway activation. It is important to emphasize that our findings are derived from an experimental animal model and should not be directly extrapolated to clinical practice. Systemic corticosteroid therapy remains the evidence-based standard for acute Bell’s palsy. The results presented here may inform the design of future clinical studies investigating acupuncture as a potential adjunctive therapy, but they should not be interpreted as evidence of clinical efficacy.

## Data Availability

The original data presented in this study are included in the article material. Further inquiries can be directed to the corresponding author.
